# Thermal Behaviour of Sm_0.5_*R*_0.5_FeO_3_ (*R* = Pr, Nd) Probed by High-Resolution X-ray Synchrotron Powder Diffraction

**DOI:** 10.1186/s11671-016-1328-6

**Published:** 2016-02-27

**Authors:** Olena Pavlovska, Leonid Vasylechko, Oleh Buryy

**Affiliations:** Lviv Polytechnic National University, 12 Bandera Street, 79013 Lviv, Ukraine

**Keywords:** Mixed rare earth ferrites, Perovskites, Crystal structure, Thermal expansion, Magnetoelastic coupling, 61, 61.05.cp, 65.40.De

## Abstract

Mixed ferrites Sm_0.5_Pr_0.5_FeO_3_ and Sm_0.5_Nd_0.5_FeO_3_ with orthorhombic perovskite structure isotypic with GdFeO_3_ were synthesized by solid-state reaction technique in air at 1473 K. Structural parameters obtained at room temperature prove a formation of continuous solid solutions in the SmFeO_3_–PrFeO_3_ and SmFeO_3_–NdFeO_3_ pseudo-binary systems. Sm_0.5_Pr_0.5_FeO_3_ and Sm_0.5_Nd_0.5_FeO_3_ show strongly anisotropic nonlinear thermal expansion: thermal expansion in the *b* direction is twice lower than in the *a* and *c* directions. The average linear thermal expansion coefficients of Sm_0.5_Pr_0.5_FeO_3_ and Sm_0.5_Nd_0.5_FeO_3_ in the temperature range of 298–1173 K are in the limits of (9.0–11.1) × 10^−6^ K^−1^, which is close to the values reported for the parent *R*FeO_3_ compounds. Subtle anomalies in the lattice expansion of Sm_0.5_Pr_0.5_FeO_3_ and Sm_0.5_Nd_0.5_FeO_3_ detected at 650–750 K reflect magnetoelastic coupling at the magnetic ordering temperature *T*_N_.

## Background

Complex oxides with perovskite structure *R*FeO_3_, where *R* is the rare earth(RE), represent an important class of functional materials. The *R*FeO_3_-based materials are used as electrodes in solid oxide fuel cells, as catalysts, gas sensory materials and semiconductor ceramics [1–6]. Complementary, the interest in the rare earth ferrites is stimulated by their interesting fundamental physical properties, such as spin-reorientation transitions at 80–480 K and the para- to antiferromagnetic transitions at 620–750 K [7–10]. Just recently, the interest to RE ferrite perovskites was renewed due to reported multiferroic properties of NdFeO_3_, SmFeO_3_ and other *R*FeO_3_ compounds [11–13]. At room temperature (RT), all RE orthoferrites adopt orthorhombic perovskite structure isotypic with GdFeO_3_ [14, 15]. No structural phase transitions were reported in the literature for *R*FeO_3_ compounds, with an exception of LaFeO_3_, which undergoes a high-temperature (HT) transition to rhombohedral structure at 1220–1280 K [16, 17]. Orthorhombic *R*FeO_3_ perovskites show strongly anisotropic thermal expansion: the expansivity in the *b* direction in the *Pbnm* setting is ca. two times lower than in the *a* and *c* directions. Subtle anomalies in the lattice expansion of PrFeO_3_ and SmFeO_3_ are observed in the *b* direction at 600–800 K, which is indicative for magnetoelastic coupling at the magnetic ordering temperature *T*_N_ [18, 19]. In ref. [9], it was shown that the spin-reorientation transition in NdFeO_3_ between 100 and 200 K is associated with changes of the *b*-lattice parameter, which has a broad local minimum in the spin-reorientation region near 160 K. However, no lattice anomalies in NdFeO_3_ were found around the Néel temperature of 687 K in [10].

The aim of the present work is the detail study of the thermal behaviour of Sm_0.5_Pr_0.5_FeO_3_ and Sm_0.5_Nd_0.5_FeO_3_ in order to reveal the possible magnetoelastic coupling in these mixed perovskite ferrites.

## Methods

Polycrystalline samples with nominal compositions Sm_0.5_Pr_0.5_FeO_3_ and Sm_0.5_Nd_0.5_FeO_3_ have been prepared from stoichiometric amounts of constituent oxides Sm_2_O_3_, Pr_6_O_11_, Nd_2_O_3_ and Fe_2_O_3_ by solid-state reaction technique according to the following reaction schemes:

$$ \begin{array}{l}3/2\mathrm{S}{\mathrm{m}}_2{\mathrm{O}}_3 + 1/2\mathrm{P}{\mathrm{r}}_6{\mathrm{O}}_{11} + 3\mathrm{F}{\mathrm{e}}_2{\mathrm{O}}_3\to\ 6\mathrm{S}{\mathrm{m}}_{0.5}\mathrm{P}{\mathrm{r}}_{0.5}\mathrm{F}\mathrm{e}{\mathrm{O}}_3 + 1/2{\mathrm{O}}_2\uparrow \hfill \\ {}1/2{\mathrm{Sm}}_2{\mathrm{O}}_3 + 1/2{\mathrm{Nd}}_2{\mathrm{O}}_3 + {\mathrm{Fe}}_2{\mathrm{O}}_3\to\ 2{\mathrm{Sm}}_{0.5}{\mathrm{Nd}}_{0.5}{\mathrm{Fe}\mathrm{O}}_3.\hfill \end{array} $$

Precursor oxides were ball-milled in ethanol for 5 h, dried, pressed into pellets and annealed in air at 1473 K for 20 h. The as-obtained product was repeatedly re-grinded and annealed at 1473 K for 20 h and, after that, slowly cooled to RT for 20 h.

X-ray phase and structural characterization of the samples was performed at room temperature by using imaging plate Guinier camera G670 (Cu K_α1_ radiation, *λ* = 1.54056 Å). Thermal behaviour of Sm_0.5_Pr_0.5_FeO_3_ and Sm_0.5_Nd_0.5_FeO_3_ structures has been studied in situ in the temperature range of 298–1173 K by means of high-resolution X-ray synchrotron powder diffraction technique. The corresponding experimental powder diffraction patterns were collected with the temperature steps of 30 K at beamline B2 of synchrotron laboratory HASYLAB/DESY (Hamburg, Germany). Structural parameters of the samples were derived from the experimental diffractograms by using full-profile Rietveld refinement technique applying WinCSD program package [20].

## Results and Discussion

**Fig. 1:**
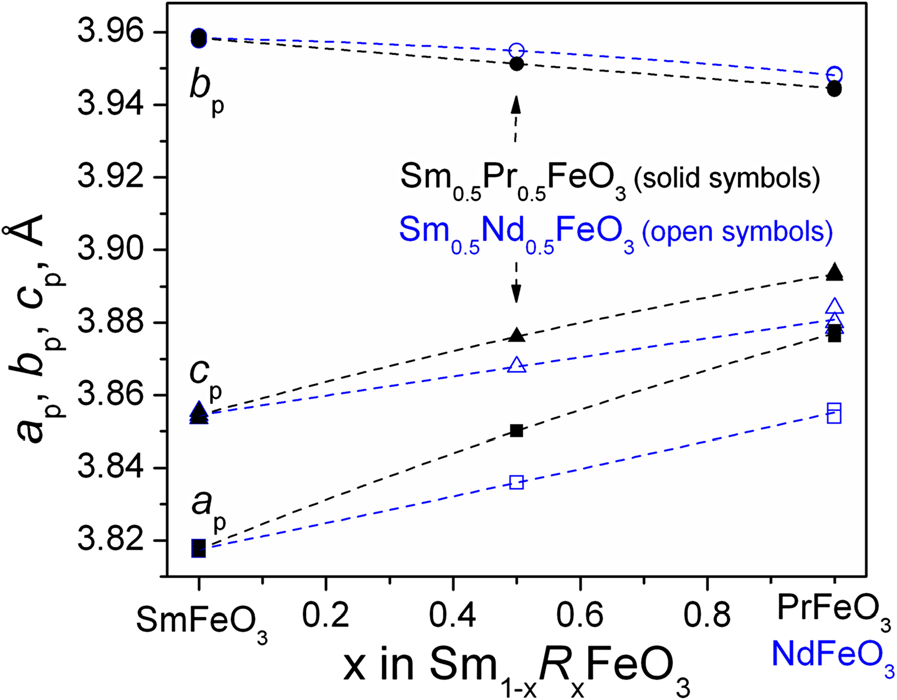
Concentration dependence of the unit-cell dimensions in the SmFeO_3_–PrFeO_3_ and SmFeO_3_–NdFeO_3_ systems. The orthorhombic lattice parameters are normalized to the perovskite cell as follows: *a*_*p*_ = *a*_*o*_/√2, *b*_*p*_ = *b*_*o*_/√2, and *c*_*p*_ = *c*_*o*_/2

X-ray powder diffraction examination revealed that both samples synthesized possess orthorhombic perovskite structure isotypic with GdFeO_3_. No extra crystalline phases were found. The unit-cell dimensions of Sm_0.5_Pr_0.5_FeO_3_ and Sm_0.5_Nd_0.5_FeO_3_ at room temperature are in good agreement with the structural data of the parent SmFeO_3_, PrFeO_3_ and NdFeO_3_ [14, 15] compounds, (Fig. 1), thus proving possible formation of continuous solid solutions Sm_1 − *x*_Pr_*x*_FeO_3_ and Sm_1 - *x*_Nd_*x*_FeO_3_ in the SmFeO_3_–PrFeO_3_ and SmFeO_3_–NdFeO_3_ systems.

**Fig. 2:**
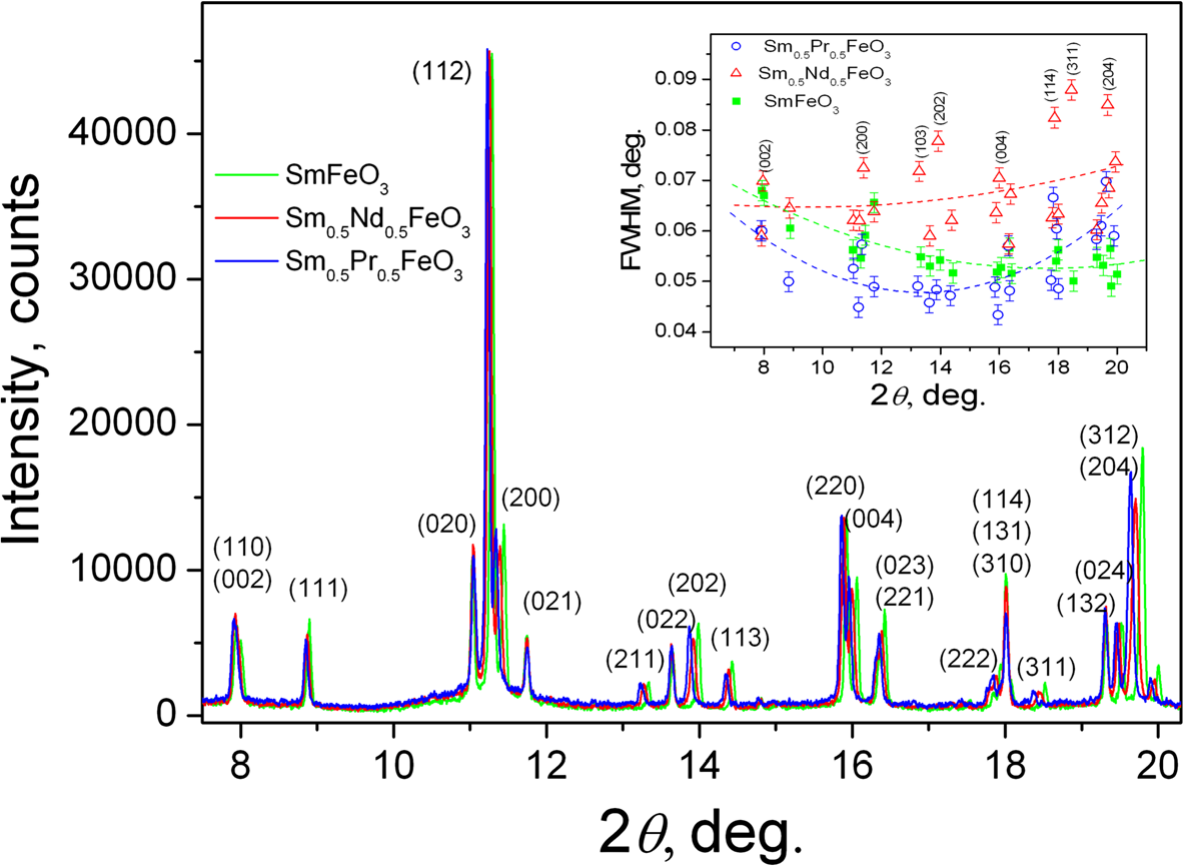
X-ray synchrotron powder diffraction patterns of Sm_0.5_Pr_0.5_FeO_3_ and Sm_0.5_Nd_0.5_FeO_3_ (*λ* = 0.53833 Å). For a comparison, the corresponding pattern of the “pure” SmFeO_3_ is given as well. *Inset* shows the angular dependence of HWFM for all three samples

Precise high-resolution X-ray synchrotron powder diffraction examination confirms phase purity of the Sm_0.5_Pr_0.5_FeO_3_ and Sm_0.5_Nd_0.5_FeO_3_ samples (Fig. 2). The values of full width at half maximum (FWHM) of the mixed samarium-praseodymium and samarium-neodymium ferrites are in the limits of 0.043°–0.089°, which is comparable with those of the “pure” SmFeO_3_ ferrite (Fig. 2, inset). Angular dependence of FWHM of Sm_0.5_Pr_0.5_FeO_3_ substantially resembles the behaviour of the parent SmFeO_3_ compound, whereas a rather scattered behaviour is observed for the Sm_0.5_Nd_0.5_FeO_3_ sample (Fig. 2, inset). To some extent, *hkl*-dependent anisotropic broadening of Bragg peaks points on the possible compositional, thermal and elastic microstrains presented in the Sm_0.5_Nd_0.5_FeO_3_ sample [21].

**Fig. 3:**
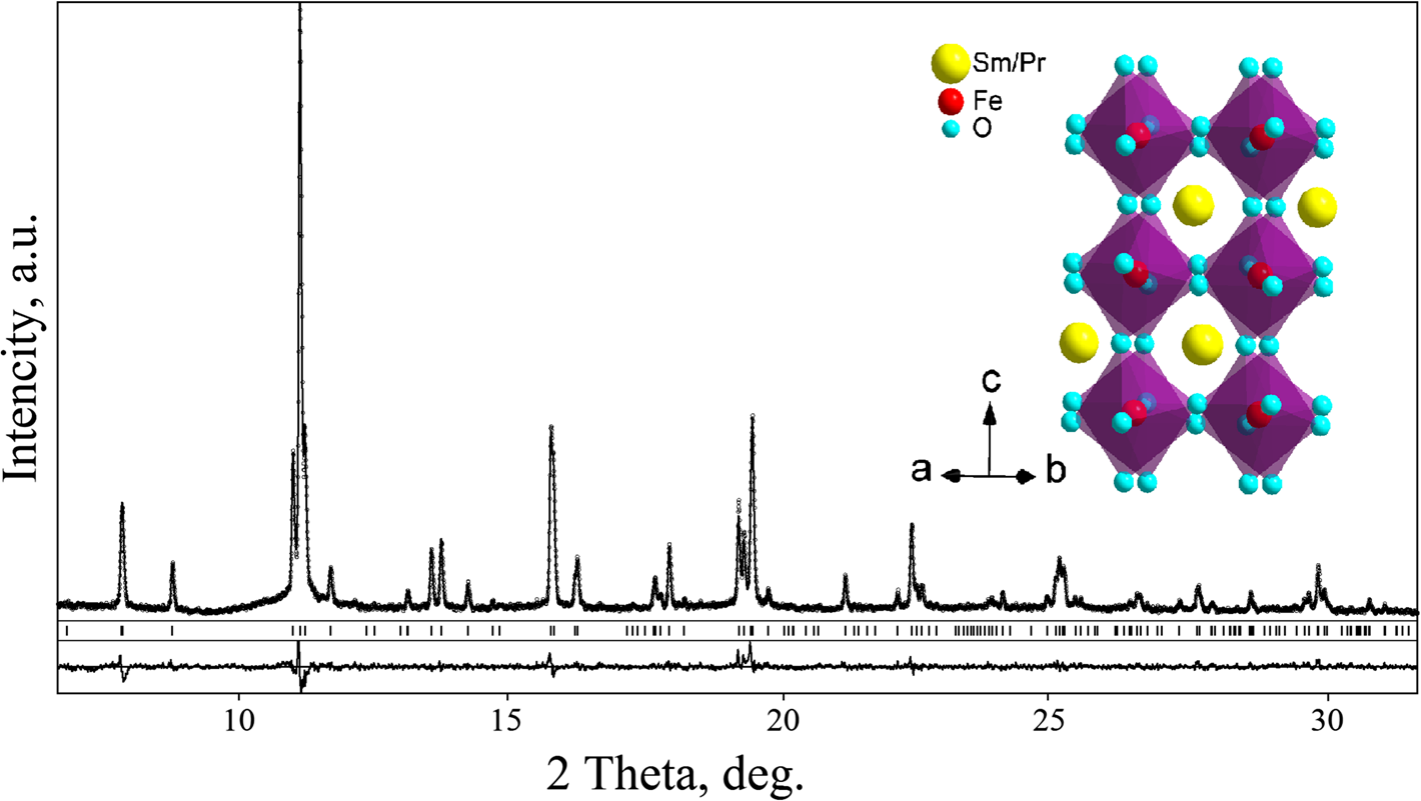
Graphical results of Rietveld refinement of the Sm_0.5_Pr_0.5_FeO_3_ structure at 1173 K. Experimental X-ray synchrotron powder diffraction pattern (*λ* = 0.53833 Å) collected at 1173 K (*dots*) is shown in comparison with the calculated pattern (*lines*). The difference between measured and calculated profiles is shown as a *curve below the diagrams. Short vertical bars* indicate the positions of diffraction maxima in the space group *Pbnm. Inset* shows the view of the structure as corner-shared FeO_6_ octahedra with Sm/Pr species located between them

In situ high-temperature X-ray synchrotron powder diffraction investigations prove that Sm_0.5_Pr_0.5_FeO_3_ and Sm_0.5_Nd_0.5_FeO_3_ remain orthorhombic at least up to 1173 K. No structural phase transitions were detected in the whole temperature range investigated. Based on the experimental X-ray synchrotron powder diffraction data, the unit-cell dimensions and positional and displacement parameters of atoms in the Sm_0.5_Pr_0.5_FeO_3_ and Sm_0.5_Nd_0.5_FeO_3_ structures between RT and 1173 K were derived by full-profile Rietveld refinement technique. As an example, Fig. 3 represents the graphical results of Rietveld refinement of the Sm_0.5_Pr_0.5_FeO_3_ structure at 1173 K. Refined structural parameters of Sm_0.5_Pr_0.5_FeO_3_ and Sm_0.5_Nd_0.5_FeO_3_ at the selected temperatures are presented in Table 1.

**Table 1:** Lattice parameters, coordinates and displacement parameters of atoms in the Sm_0.5_Pr_0.5_FeO_3_ and Sm_0.5_Nd_0.5_FeO_3_ structures at RT, 753 and 1173 K

Atoms, sites	Parameters, residuals	Sm_0.5_Pr_0.5_FeO_3_			Sm_0.5_Nd_0.5_FeO_3_		
		*T* = 298 K	*T* = 753 K	*T* = 1173 K	*T* = 298 K	*T* = 753 K	*T* = 1173 K
	*a*, Å	5.4449(1)	5.4749(1)	5.5064(2)	5.4248(1)	5.4554(2)	5.4872(2)
	*c*, Å	5.5879(1)	5.6038(1)	5.6165(2)	5.5930(1)	5.6093(2)	5.6228(2)
	*b*, Å	7.7523(2)	7.7962(2)	7.8378(2)	7.7357(2)	7.7795(2)	7.8210(3)
	*V*, Å^3^	235.87(2)	239.19(2)	242.39(2)	234.70(2)	238.06(2)	241.31(3)
Sm/Pr(Nd), 4*c*	*x*	−0.0099(3)	−0.0086(4)	−0.0081(4)	−0.0095(3)	−0.0089(4)	−0.0072(5)
	*y*	0.0495(2)	0.0466(2)	0.0433(2)	0.0517(2)	0.0497(2)	0.0451(2)
	*z*	1/4	1/4	1/4	1/4	1/4	1/4
	*B*_iso_, Å^2^	0.98(1)	1.12(2)	1.59(2)	0.942(9)	1.13(2)	1.58(2)
Fe, 4*b*	*x*	0	0	0	0	0	0
	*y*	1/2	1/2	1/2	1/2	1/2	1/2
	*z*	0	0	0	0	0	0
	*B*_iso_, Å^2^	0.76(3)	0.75(4)	1.08(5)	0.83(3)	0.70(4)	1.16(5)
O1, 4*c*	*x*	0.092(2)	0.091(2)	0.094(2)	0.090(2)	0.084(2)	0.085(2)
	*y*	0.481(2)	0.484(2)	0.481(2)	0.4821(14)	0.487(2)	0.482(2)
	*z*	1/4	1/4	1/4	1/4	1/4	1/4
	*B*_iso_, Å^2^	0.7(2)	0.9(3)	1.5(3)	0.5(2)	0.6(3)	1.7(3)
O2, 8*d*	*x*	−0.2934(13)	−0.2955(15)	−0.296(2)	−0.2961(13)	−0.299(2)	−0.289(2)
	*y*	0.2939(13)	0.2960(14)	0.301(2)	0.2965(12)	0.297(2)	0.294(2)
	*z*	0.0433(10)	0.0403(11)	0.0362(13)	0.0465(9)	0.0454(11)	0.0462(13)
	*B*_iso_, Å^2^	0.39(12)	0.4(2)	0.6(2)	0.44(12)	0.9(2)	0.6(2)
	*R*_I_	0.104	0.102	0.109	0.102	0.104	0.104
	*R*_P_	0.168	0.170	0.185	0.166	0.187	0.196

**Fig. 4:**
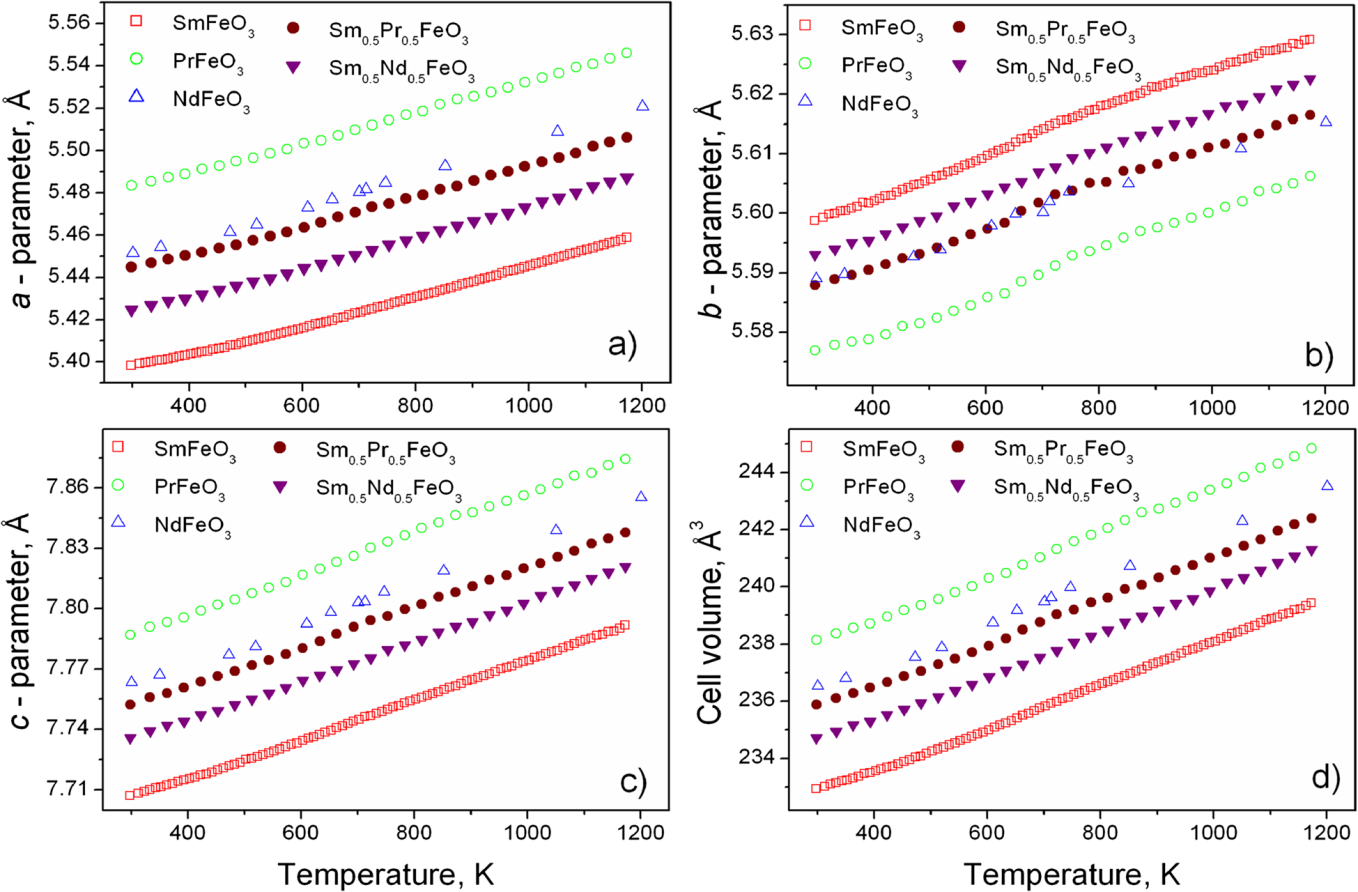
Temperature dependencies of the lattice parameters (**a**-**c**) and unit cell volumes (**d**) of Sm_0.5_Pr_0.5_FeO_3_ and Sm_0.5_Nd_0.5_FeO_3_. Thermal evolution of the unit cell dimensions of SmFeO_3_ [19], PrFeO_3_ [18] and NdFeO_3_ [10] is shown for a comparison

Temperature dependencies of the unit-cell dimensions of Sm_0.5_Pr_0.5_FeO_3_ and Sm_0.5_Nd_0.5_FeO_3_ in comparison with the literature data for the “pure” ferrite perovskites SmFeO_3_ [19], PrFeO_3_ [18] and NdFeO_3_ [10] are presented in Fig. 4.

Temperature evolution of the lattice parameters of mixed Sm-Pr and Sm-Nd ferrites resemble for the most part the thermal behaviour of the parent compounds. In both cases, clear deviations from the “normal” trend are observed in the *b* direction at 650–750 K, whereas much less visible anomalies in the lattice expansion are observed in the *a* and *c* directions (Fig. 4a–c). It is evident that similar to SmFeO_3_ and PrFeO_3_, a kink in the *b*-lattice expansion of Sm_0.5_Pr_0.5_FeO_3_ and Sm_0.5_Nd_0.5_FeO_3_ is associated with the para- to antiferromagnetic transitions that occurred in these specimens at the Néel temperatures. Earlier, nonlinear lattice expansion across the antiferromagnetic to paramagnetic transitions was also observed in LaFeO_3_ at *T*_N_ = 735 K [17].

**Fig. 5:**
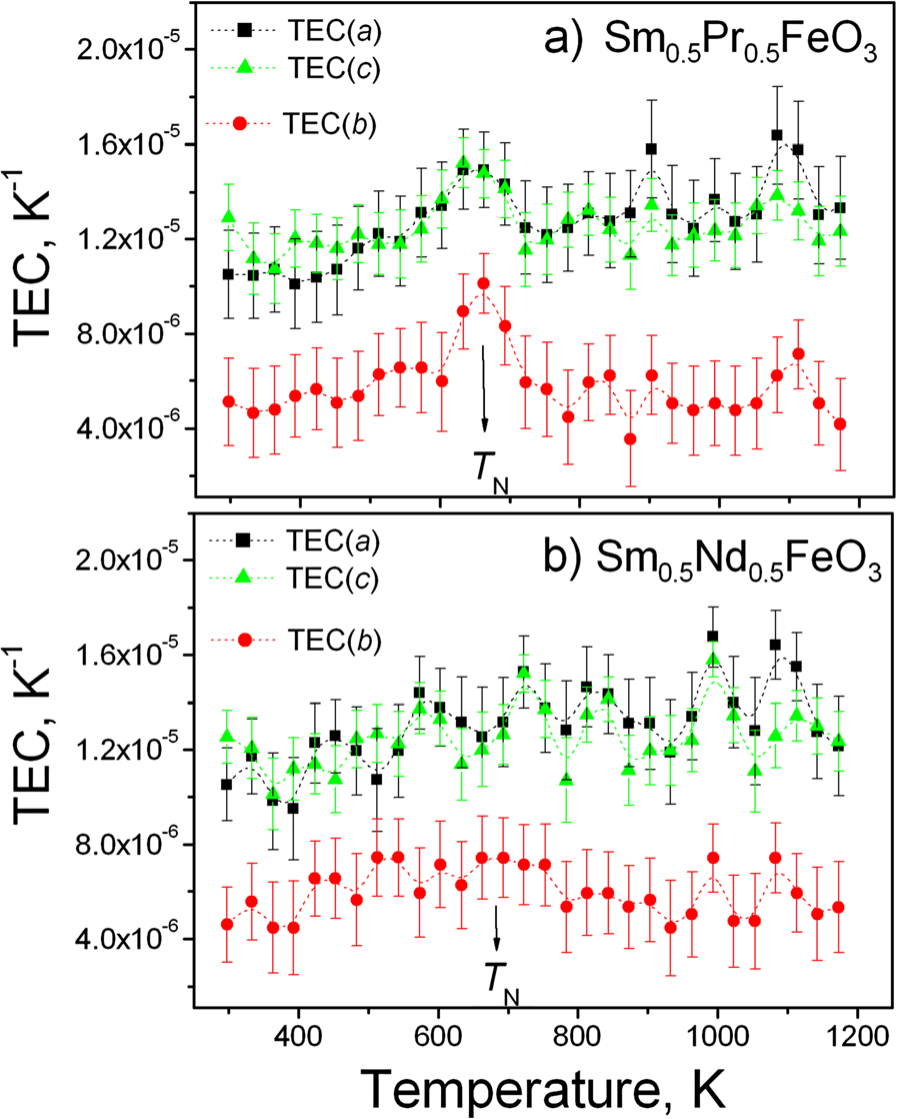
Temperature evolution of the linear thermal expansion coefficients of Sm_0.5_Pr_0.5_FeO_3_ (**a**) and Sm_0.5_Nd_0.5_FeO_3_ (**b**) The corresponding values of TECs in different crystallographic directions are derived by differentiation of the experimental values of the unit-cell dimensions in the temperature range of 298–1173 K. The *dashed lines* are quid for the eyes

Similar to the “pure” *R*FeO_3_ perovskites, thermal expansion of Sm_0.5_Pr_0.5_FeO_3_ and Sm_0.5_Nd_0.5_FeO_3_ shows a clear anisotropic behaviour. Calculated thermal expansion coefficients (TECs) in the *b* direction are in the limits of (5.3–6.2) × 10^−6^ K^−1^ which is twice lower than the values of (11.1–13.6) × 10^−6^ K^−1^ in the *a* and *c* directions (Fig. 5). Such anisotropic thermal expansion is rather typical for the majority of perovskite oxides with a GdFeO_3_ type of structure and is inherent for the families of rare earth aluminates, gallates [22–25] and other perovskites. The average linear thermal expansion coefficients of Sm_0.5_Pr_0.5_FeO_3_ and Sm_0.5_Nd_0.5_FeO_3_ in the temperature range of 298–1173 K are in the limits of (9.0–11.1) × 10^−6^ K^−1^. It is close to the TEC value of (10.8–11.8) × 10^−6^ K^−1^ reported for LaFeO_3_ [26, 27] and other rare earth ferrites confirming the suggestion that the nature of rare earth ions does not influence the thermal expansion in *R*FeO_3_ [15].

Subtle maxima at the TEC curves of Sm_0.5_Pr_0.5_FeO_3_ around 670 K (Fig. 5a) reflect the observed lattice anomalies at the Néel temperature. In spite of no obvious maxima observed on the TEC curves of Sm_0.5_Nd_0.5_FeO_3_, a change of the slope of the TEC(*b*) values occurs around 650–700 K (Fig. 5b). A similar step at the thermal expansion coefficient at 723 ± 50 K, corresponding with the Néel temperature, has been revealed in LaFeO_3_ by dilatometric measurements [16].

The lattice expansion of Sm_0.5_Pr_0.5_FeO_3_ and Sm_0.5_Nd_0.5_FeO_3_ could be also affected by a possible change of the oxygen defect structure during the heating of the samples, as it was detected in PrFeO_3_ and SmFeO_3_ by thermogravimetric measurements [15]. As it was shown, detectable weight loss due to the fast oxygen desorption begins in these ferrites above 573 K. As a consequence, thermal expansion behaviour of SmFeO_3_ shows a change of the slope at around 593 K close to the temperature of sharp weight loss detected by TGA [15].

## Conclusions

Crystal structure parameters of the mixed samarium-praseodymium and samarium-neodymium ferrites Sm_0.5_Pr_0.5_FeO_3_ and Sm_0.5_Nd_0.5_FeO_3_ synthesized by solid-state reaction technique in air at 1473 K have been studied in a wide temperature range of 298–1173 K by means of high-resolution X-ray synchrotron powder diffraction technique. Close analysis of the temperature dependence of the unit-cell dimensions in comparison with the literature data for the parent *R*FeO_3_ compounds revealed strongly anisotropic lattice expansion and subtle anomalies associated with the para- to antiferromagnetic transitions at 650–750 K. The average linear thermal expansion coefficients of Sm_0.5_Pr_0.5_FeO_3_ and Sm_0.5_Nd_0.5_FeO_3_ derived from the experimental values of the unit-cell dimensions in the temperature range of 298–1173 K are in the limits of (9.0–11.1) × 10^−6^ K^−1^, which is close to the corresponding values reported for the parent *R*FeO_3_ compounds.
